# Bioreactor-Based Tumor Tissue Engineering

**Published:** 2016

**Authors:** A.E. Guller, P.N. Grebenyuk, A.B. Shekhter, A.V. Zvyagin, S. M. Deyev

**Affiliations:** Macquarie University, Sydney, 2109, New South Wales, Australia; ARC Centre of Excellence for Nanoscale BioPhotonics, Macquarie University, Sydney 2109, New South Wales, Australia; Sechenov First Moscow State Medical University, Institute for Regenerative Medicine, 8, Trubetskaya Str., Moscow, 119992, Russia; Lobachevsky Nizhniy Novgorod State University, 23, Gagarina Ave., Nizhniy Novgorod, 603950, Russia; Inetex LTD, 10, Plaut Str., Rehovot, 76706, Israel; Institute of Bioorganic Chemistry, 16/10, Miklukho-Maklaya Str., Moscow, 117871, Russia; National Research Tomsk Polytechnic University, 30, Lenina Ave., Tomsk, 634050, Russia

**Keywords:** bioreactors, cancer, models, tissue engineering

## Abstract

This review focuses on modeling of cancer tumors using tissue engineering technology. Tumor tissue engineering (TTE) is a new method of three-dimensional (3D) simulation of malignant neoplasms. Design and development of complex tissue engineering constructs (TECs) that include cancer cells, cell-bearing scaffolds acting as the extracellular matrix, and other components of the tumor microenvironment is at the core of this approach. Although TECs can be transplanted into laboratory animals, the specific aim of TTE is the most realistic reproduction and long-term maintenance of the simulated tumor properties *in vitro *for cancer biology research and for the development of new methods of diagnosis and treatment of malignant neoplasms. Successful implementation of this challenging idea depends on bioreactor technology, which will enable optimization of culture conditions and control of tumor TECs development. In this review, we analyze the most popular bioreactor types in TTE and the emerging applications.

## INTRODUCTION

An *in vitro *cell or tissue culture is a traditional instrument of research in the field of cancer biology and development of new methods for the prevention, diagnosis, and treatment of this disease. Primary and linear cells of human and animal tumors represent a convenient model for studying the molecular and cellular mechanisms of malignant growth and for evaluating drug effects. However, about 95% of drugs which exhibit significant antitumor effects in experiments in cell cultures and in laboratory animals demonstrate insufficient efficacy or unacceptable toxicity in clinical trials [1]. A possible and likely explanation for this is the poor relevance of existing *in vitro *and *in vivo *cancer models to human tumors that have a dynamic and complex structure and heterogeneous cell composition [1-3].

**Fig. 1:**
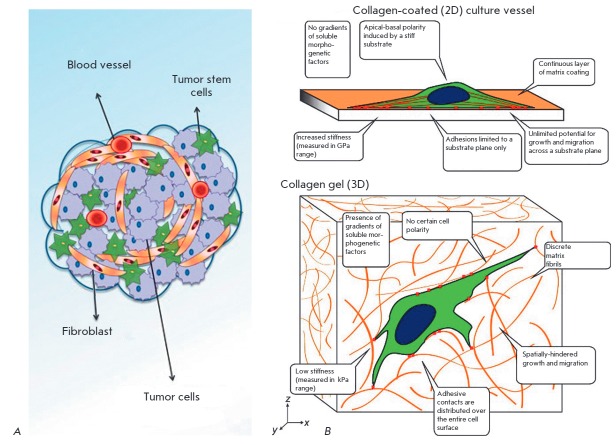
Malignant tumor structure (*A*) (a schematic view, adapted from [8])) and conditions for traditional 2D-tissue culture* in vitro *(*B*) (adapted from [9]). (*A*) The tumor is a 3D-structure. Due to abnormal local blood circulation and innervation, the tumor possesses multiple metabolic gradients which contribute to the genetic instability of malignant cells. Phenotype selection affects the dynamic responses of a cancer stem cell pool. In addition to the neoplastic cell population, resident cells of the affected organ and cells of the inflammatory infiltrate (including macrophages, lymphocytes, eosinophils, and sometimes plasma cells) are involved in the tumor. The extracellular matrix, blood vessels, and connective tissue inclusions are the second component, known as the stroma of the tumor. The degree of stroma development in malignant tumors varies notably and significantly affects the course of the disease and tumor drug resistance. Also, sites with active growth, necrotic zones, hemorrhages, and purulent pockets can occur within the tumor. (*B*) Changes observed in a 2D culture are induced by the selection of specific cellular phenotypes and abnormal interactions between cells and their micro- and macro-environments.

The most important factors associated with experiments in conventional monolayer cell cultures (2D) include the selection of a specific cell phenotype (adapted for growth on a plastic surface) from an initially very heterogeneous tumor cell population, abnormal cell polarization resulting from limited exposure of the cell surface to the culture medium, a drastic reduction in the number of cell-cell contacts, and a lack of cell-matrix interactions and metabolic gradients [4-6]. Together, these factors make a 2D-culture inadequate for capturing the critical mechanisms in cancer biology [7], such as the heterogeneity of tumor cell populations, as well as the intensive interaction between the tumor and its microenvironment and the whole organism (*Fig. 1*).

**Fig. 2:**
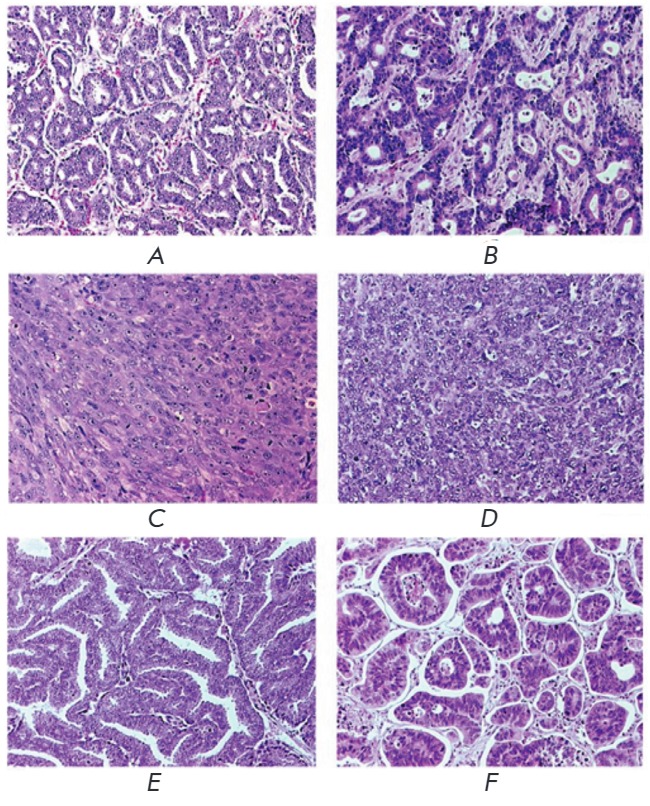
Comparisons of histological structures of prostate (*A, C, E*) and colon (*B, D, F*) cancers observed in primary tumors (*A, B*) and the cancers propagated as model systems in mice (*C-F*). Tumors obtained by subcutaneous engraftment of suspensions of linear cells PC-3M (*C*) and Colo205 (*D*) have a homogenous structure with absence of specific glandular elements formed by cancer cells and the lack of a stromal component. Significant alterations of tumor-stroma ratio are also notable in the cases of subcutaneous grafting of surgical biopsy specimens of original human primary tumors (*E, F*). Adapted from [12] with changes.

Cancer models in laboratory animals also have some notable disadvantages. For example, in simulations of human tumors in mice by implanting cellular allografts, which is one of the most popular approaches, the histological features of human neoplasms are reproduced inaccurately or not reproduced at all (*Fig. 2A-D*). In addition, the lifespan of laboratory animals is oftentimes shorter than the period of metastases development [8]. Xenografts derived from the tumor tissue of patients and transplanted into mice with a suppressed immune system (nude, SCID) represent a realistic model of human tumors [10, 11]. These approaches reflect the structure and function of human tumor at the tissue level adequately to some extent (*Fig. 2E,F*), while the host organism plays the same role as that of the culture medium in *in vitro* cultures. At the same time, the physiology of athymic or SCID mice significantly differs from that of humans. The high cost and low reproducibility of these models limit their applications.

Aiming to more accurately reproduce the histological structure of tumors and their physiological properties, technologies of co-culturing of different cell types and three-dimensional (3D) tumor models have been introduced. The latter include multicellular spheroids and cancer cell cultures on matrices of various compositions and structures (gel, fibrous, etc.). One of the most promising approaches is tumor tissue engineering (TTE), which is a new method of 3D-modeling of malignant neoplasms based on the production of complex constructs, including malignant cells, solid porous or fibrous cellular carriers (scaffolds), acting as an extracellular matrix, and other components of the tumor microenvironment. Tissue-engineered tumor models (TETMs) are designed for studying cancer biology and the development of methods for the diagnosis and treatment of malignant tumors. The basic principles of TTE, its advantages and limitations, as well as the implemented models, were discussed in detail in recent reviews [8, 13-19].

As its name suggests, TTE makes use of the tissue engineering (TE) technology of normal tissues in terms of a combination of certain cells and scaffolds with subsequent control of the produced tissue engineering constructs (TECs) [20]. At the same time, TTE is meant for research, unlike TECs of normal tissues, which are used for therapeutic purposes. In general, a tissue engineering model of healthy tissue is a 3D-culture of normal cells on a scaffold, the TEC, that is “assembled” and matures *in vitro *and then is implanted into a patient organism to replace damaged or lost tissues or organs. Then, engraftment of the reconstructed structure occurs to ensure the viability of the structure and its functionality. TECs are often used in regenerative medicine and serve as a temporal functional tissue or organ prostheses that is expected to be bioresorbed up to complete replacement with the organism’s own tissues. In contrast, cancer TECs include primarily malignant cells able to survive for a long time outside the body, preserving a structural and functional similarity to the simulated tumors even under *in vitro *conditions. Tumor TECs can also be implanted into laboratory animals, e.g., to study the angiogenic, invasive, and metastatic potential of the engineered tumors. However, the use of these bioartificial tissues *in vitro *seems to be most attractive for improvement of the reproducibility of the results, development of high-throughput test systems for pharmacological research, and for the replacement of animals in research.

The differences in the growth, differentiation, and metabolism rates between normal and cancer cells obviate a key problem of regenerative medicine: the expansion of the cell population within a TEC (e.g., during controlled differentiation of stem cells). On the other hand, these call for the development of new methods and systems of 3D culture that enable the formation and maintenance of bulky and metabolically active tissue structures outside the body; i.e., in the absence of normal homeostatic systems. Similar problems are partially addressed in the modern systems for the temporary storage and maintenance of donor organs.

In tissue engineering, growth and maintenance of TECs occurs in bioreactors (BRs) until implantation [21]. To this aim, purpose-designed systems are needed to automate cell and tissue culturing processes *in vitro* and provide optimal physico-chemical conditions for TEC development. The purpose of this review is to analyze the current state of bioreactor-based tissue engineering modeling of malignant tumors.

## TUMOR TEC COMPONENTS

**Fig. 3:**
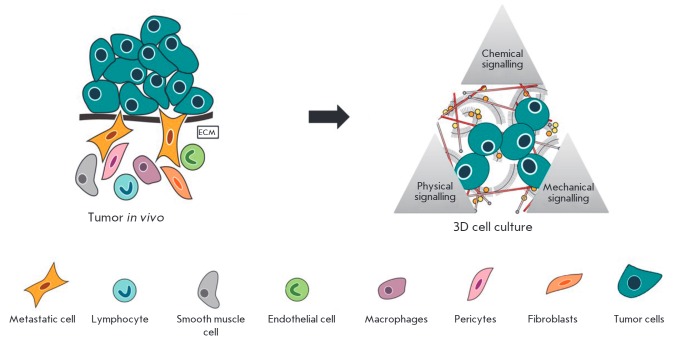
Principles of formation of tumor TECs. In order to create biomimetic tumor TECs the key components of the original tumor (as cancer cells and a scaffold, representing the extracellular matrix) should be included into the model. In addition, it is very important to reproduce the conditions of tumor growth by the inclusion of physical and chemical signalling factors. Adapted from [23] with changes.

Cells and scaffolds are the main components of TECs (*Fig. 3*). Cells can be presented by one or several types simultaneously (e.g., by fibroblasts and hepatocytes in liver models); however, the tissue specificity of TECs is determined by the most abundant cellular population. In particular, the cellular component of tumor TECs can be formed by both the primary cells isolated from tumor biopsy fragments (from a primary or metastatic foci) or the linear cancer cells obtained by using a special selection and culture procedures. Cells with a varying degree of differentiation and with different metastatic potentials can be selected. In addition to tumor cellular populations, TECs can also include stromal elements (fibroblasts, pericytes and endothelial and smooth muscle cells), the main cells of a resident organ (e.g., hepatocytes in models of liver tumors or osteoblasts and bone marrow cells in the investigation of neoplastic processes in bones), and cells of an inflammatory infiltrate (macrophages, lymphocytes, neutrophils, plasma cells, eosinophils) [22].

Scaffolds represent an important component of TEC. They function as bioactive extracellular matrices serving to provide mechanical support for the cells and promote cellular adhesion and motility (which switches to a number of signaling pathways sensitive to the cytoskeletal organization), provide mechanical and biochemical integration of the construct, stimulate the required differentiation (in TECs of normal tissues), or maintain a specified phenotype and functional activity of the cells. The scaffold architecture ensures the formation of the gradients of signaling molecules and oxygen within TECs and enables the study of the role of cell-matrix interactions in the regulation of carcinogenesis. The convoluted and interdependent effects of mechanical factors, nanotopography, matrix geometry, and cell adhesion are also investigated in the framework of TEC [24].

Scaffolds can be produced using fibrous and porous materials made from synthetic polymers (e.g., polylactate, polycaprolactone, polylactoglycolide) or materials of natural origin (collagen, chitosan, hyaluronic acid) [17], as well as specially treated natural tissues and organs [25, 26]. The major advantage of synthetic scaffolds fabricated by engineering methods (electrospinning, 3D printing, etc.) is a high degree of chemical composition certainty and precise control over spatial organization and mechanical properties, which allows one to study the influence of single signaling factors on tissue morphogenesis. However, such scaffolds do not maintain the necessary adhesion and long-term proliferation of cells, with a few exceptions. Furthermore, they mimic the modeled original tissue poorly, largely remaining a 3D-analogue of conventional plastic culture dishes. Scaffolds made of natural polymers have high biocompatibility, although strict control of their composition, geometry, and biomechanical properties is challenging [17].

An alternative approach involves the processing of natural tissues or organs by removing their cellular elements while preserving the composition and structure of the extracellular matrix. This process is called decellularization (DCL). It results in the production of scaffolds, which are known as decellularized tissues or acellular matrices, tissues, or organs (DCL matrix, DCL tissue, DCL organ, respectively) [26, 27]. Therefore, DCL allows one to prepare scaffolds that reproduce the natural microenvironment of cells in a tissue or organ very closely. Modern DCL methods result not only in scaffolds containing the major extracellular matrix components, such as collagen and elastic fibers, but also templates maintaining the integrity of the basal membranes of blood vessels, which ensures the presence of integrated vascular conduits (decellularized walls of blood vessels of various calibers) that can later be used to perfuse the bioengineered tissue. This feature is of great importance, because the nutrition of the inner regions of TECs is a challenge in tissue engineering and it is critical for TTE.

## MAIN OBSTACLES ASSOCIATED WITH “ASSEMBLY” AND CULTURE OF ENGINEERED TUMOR TISSUES THAT REQUIRE THE USE OF BIOREACTORS

Bioreactors (BRs) are closed systems where biological processes occur under strictly controlled conditions [28]. The concept of BRs (as chemostats or fermenters) has been used for growing microbial cultures and obtaining various products of cells for a long time. A typical bioreactor system includes a tank isolated from the environment (flask, vessel, chamber), the actuator components (pumps, motors, etc.), sensors, and, very often, special controllers and software for managing and monitoring the biotechnological process. BRs designed for TE have been used for growing cells and TECs, as well as for exploring the effects of biochemical and biomechanical factors on the development of cells and tissues. There are several key difficulties related to the assembly of tumor TECs and their further *in vitro *culture. An optimal solution for these problems requires the use of bioreactor technologies.

**Expansion of cellular populations**

A TETM size can vary from a few cubic millimeters to a whole organ of the human or animal body, but the number of necessary cells always largely exceeds the population of a typical monolayer cell culture. Therefore, the first step in the development of a tumor TEC is to expand the number of cells of the required types. This is possible only with the use of a large surface area for their growth. Co-cultivation of several cell types often involves simultaneous expansion of the cells in different conditions. In some cases, the cell populations used to create TECs are prepared as multicellular spheroids requiring special cultivation conditions. Automation and improved control over the cell culture processes afforded by BRs is instrumental in addressing these problems.

**Scaffold recellularization**

The second step in the development of TECs is recellularization (RCL), which is the colonization of 3D-scaffolds with cells – [29, 30]. The basic technique of a static culture uses dropwise seeding of cells on a scaffold. Then, the cell population spontaneously distributes through the matrix due to gravity and cell migration. However, this method is not effective enough for the preparation of complex tissues and bulky constructs as it does not ensure the uniform distribution of cells throughout the volume of the scaffold and, therefore, does not allow controlled development of the tissue.

**Nutrition and metabolism of TECs**

The third step is the delivery of the substances which are necessary for the growth and function of cells and removal of metabolic products throughout the TEC. This controlled and optimal mass transfer, in terms of its effect on a tissue, represents the most important goal of bioreactor technologies [28]. In a static culture *in vitro*, this can be achieved by periodical replacement of a culture medium. However, the latter method is suitable only for experiments with cultured objects of small volume, such as cells in a monolayer, a suspension, or thin tissue sections. It is known that the diffusion limit for oxygen in human tissues ranges from 100 μm to 200 μm [31]. As a result, the medium reaches the cells in a TEC only by diffusion in the absence of continuous stirring or pumping. Therefore, the central part of a TEC suffers from insufficient oxygen and nutrients, and removal of metabolic products. This can lead to hypoxia, acidosis, and cell death. Convection fluid flows in dynamic systems improve mass transfer and are preferred. However, agitation of a culture medium can cause damage to the cells and scaffold due to the excessive shear stress [32] associated with the uneven dynamics of the different fluid layers. Therefore, it is important to keep a balance both between the diffusion and convection transport and between the biomechanical properties and metabolic needs of the grown structures. A promising approach to solving the problem of oxygen transport within TECs is using BRs with built-in perfusion systems. Taking into account the complexity of maintenance of TETMs, it is desirable to automate the process of constant supply of a fresh culture medium to the cells and removal of the metabolic products combined. This automation is controlled by real-time monitoring of the biochemical parameters of the culture medium, followed by their feedback-informed tuning.

**Control of parameters in a BR culture chamber**

Long-term maintenance of sterility is critical for TETM. Since model tissue maturation takes several months, TEC contamination has fatal consequences. In addition, the materials of a BR culture chamber have to be biocompatible and bioinert, with no influence on the tissue being cultured. At the same time, the materials must withstand a humid environment at 37 °C and sterilization by autoclaving, radiation, or chemical treatment. A BR chamber made from transparent material enables visual monitoring and use of optical imaging of TECs [21, 33, 34].

Control of the physicochemical parameters of the environment formed in a culture chamber and management of these properties are important both for maintaining TEC viability and for simulating conditions typical for malignant tumors: e.g., acidosis, hypoxia and increased tissue pressure [35, 36]. Regulation of temperature, pH, and the gas composition of the culture chamber environment, introduction/removal of certain signaling molecules, controlled physical impact on the forming tissues (pressure, tension, bending, etc.), a special electromagnetic environment, or electrical stimulation of TECs, etc. [37] are often required.

## TYPES OF BIOREACTORS USED IN TTE

Most BRs exert their action on TECs through the culture medium. There are six BR types (*Table 1*): 1) BRs with static cultivation systems, 2) stirring BRs, 3) rotary BRs, 4) hollow-fiber BRs, 5) perfusion BRs, and 6) microfluidic BRs. In addition, there is a special class of BRs that acts on the TECs components directly, by means of mechanical, electromagnetic or other stimuli (they are discussed below in a special section).

**Table 1:** Comparative characterization of bioreactors with culture-medium-mediated action on TECs*

BR types	Conditions of use	Mass transfer mechanism	Shear stress	Specialization in relation to objectives of tumor tissue engineering	Disputable questions
Static culture systems (conventional culture vessels: plates, flasks, etc.)	Portion replacement of a culture medium	Diffusion	Very small	Expansion of cellular mass, production of multicellular spheroids	Overcoming the mass transfer limitations (e.g., creation of hybrid systems, such as perfusion plates); automation of the operations
Stirring BRs	Stirring of a culture medium with use of special agitators; shaking or rotating of the culture vessels	Convection (high)	High	DCL of tissues and organs, formation of spheroids, RCL of TECs	A balance between mass transfer and shear stress
Rotary BRs	Stirring of a culture medium by the movement of the culture chamber walls; a reduction in shear stress by creating microgravity; oxygenation of a medium through a special membrane	Convection (high)	Low	Production of spheroids and a 3D cell culture on microcarriers	Operating modes (including rotational speed), especially when growing bulky TECs
Hollow-fiber BRs	The flow of a culture medium through artificial porous semipermeable fibers mimicking the blood vessels penetrating TECs, oxygenation of a medium through a special membrane	Convection (medium) and diffusion (high)	Very low	Expansion of the cells with a high metabolic rate	Nondestructive control and extraction of TECs from BRs
Perfusion BRs	The flow of culture medium around or through a TEC, by natural or artificial vascular conduits; medium oxygenation by means of a special device	Diffusion (high) and convection (moderate)	Moderate	DCL of tissues and organs, RCL of dense scaffolds, maintenance of 3D cultures on solid scaffolds, creation of the specific cultivation conditions in accordance with experiment purposes	Optimization of perfusion parameters, RCL uniformity, seeding scaffolds with cells, cell adhesion
Microfluidic BRs	A static culture or strictly laminar flow of a culture medium directly through cell mass or TECs, or interaction of cells with the medium through semipermeable barriers/ membranes	Diffusion (high) and convection (moderate)	Adjustable	3D cultures on hydrogel scaffolds, simulation of angiogenesis and invasion of tumor cells, co-cultivation of different cell types, investigation of effects of fluid flow movement through a tissue; growth of spheroids; high-throughput screening of pharmaceuticals	Optimization of microfluidic chip design and biological validity of models

^*^Adapted from [38] with amendments.

## STATIC CULTIVATION SYSTEMS IN TTE

Historically, the first types of BRs used in tissue engineering were static cultivation systems. They include conventional Petri dishes, flasks, bottles, and plates in which the growing cells, tissues and culture media were stationary. Culture vessels can be supplemented with porous and fibrous scaffolds and, also, with special mesh inserts with a certain pore size, which enables the study of the effects of the signaling factors with a given size of molecules/carriers, as well as the migration and invasive activity of the cells. Plates with special low-adhesive coatings or “gravitational traps” were used for the development of multicellular spheroids. However, mass transfer in static cultivation systems occurred exclusively due to gravity and diffusion. A significant advantage of static BRs is their commercial availability and ease of use. These systems are especially popular in high-throughput screening of pharmaceutical compositions.

Static BRs have been used to create tissue engineering models of breast, lung, and intestine cancers, Ewing’s sarcoma, metastatic prostate cancer, and some other neoplasms (*Table 2*). However, it proved possible to maintain TECs only on membrane-like scaffolds with a thickness of less than 1 mm in the absence of active movement of the culture medium. Cell growth in the thicker matrices occurred predominantly on the scaffold surface. For example, we observed this effect during static cultivation of tumor cells and normal epithelium on 3 - 4 mm sections of a DCL organ (rabbit kidney) [39], as well as during RCL of a tubular acellular vascular matrix [40], and hybrid scaffolds [41], with buccal epithelial cells.

**Table 2:** Tissue engineered tumor models produced in static bioreactors

Tumor	Scaffold	Cells	Result	Reference
Breast cancer	DCL matrix of human adipose tissue	MCF-7, BT474, SKBR3	Phenotypic similarity to breast cancer biopsy tissues in the 3D culture on the DCL matrix is higher than that in a culture on Matrigel	[42]
	Silk fibroin	MDA-MB-231	Sensitivity to anticancer drugs in the cancer 3D model is reduced compared to that in 2D	[43]
Lung cancer, breast cancer, colorectal cancer, pancreatic cancer, ovarian teratocarcinoma, fibrocystic breast disease	DCL matrix synthesized in vitro by mouse embryonic fibroblasts (NIH3T3 line)	NCI-H460; PA-1; PA-1/E6; HCT116; HCT116/p53–; SW620; COLO 205; PANC-1; MCF7; HS 578T; MCF10A	The role and mechanisms of integrin-mediated signalling cascades in cell resistance to the action of antitumor agents (taxol) were studied. Prospects of using the cell-derived DCL scaffolds for drug testing were indicated	[44]
Lung cancer; the metastases of breast cancer, colorectal cancer and esophageal squamous cell carcinoma to the lungs	A DCL matrix of human lung cancer synthesized in vivo by lung cancer cells A549 (cell xenograft implanted in mice)	A549; MCF-7; SW-480; KYSE-510	The effect of the methods of DCL , mechanical properties and porosity of a produced matrix on the cell growth rate, cell viability, cell invasion into the matrix, and secretion of growth factors	[45]
Lung cancer metastases to the intestine	A DCL matrix of the porcine intestinal mucosa (in the form of a stretched membrane)	HCC827; A549	The superficial penetration of cells into a scaffold only was demonstrated. The effect of a 3D matrix on proliferation, apoptosis, and invasion compared to a culture in 2D was shown. The protein distribution and cell morphology in a 3D culture were similar to those in real tumors. Different cell sensitivity to gefitinib, depending on the presence of the epithelial growth factor receptor EGFR (not found in a 2D culture). The model was used to show an early stage of invasion	[46]
Ewing’s sarcoma	Porous 3D electrospun poly(ε-caprolactone) scaffolds	TC-71	Increased drug resistance of tumor cells on a 3D matrix compared to that in a monolayer culture. Significant differences between 3D and 2D cultures in expression of the insulin-like growth factor 1 (IGFR-1, the target for rapamycin)	[47]
	DCL bone seeded with human mesenchymal stem cells	HTB-10, HTB-166	Cells that lost a specific phenotype in a 2D culture restored their specific gene expression profile on a DCL matrix. Genes that may be the therapeutic targets were identified	[48]
Prostate cancer metastases to bones	A tissue engineered bone: a poly-caprolactone scaffold “wrapped up” with a sheet of osteoblasts	PC3; LNCaP	An elevated level of matrix metalloproteinases and other markers of a metastatic phenotype activation	[49]
		LNCaP (in PEG-gel)	Osteoblasts induce paracrine effects that can promote osteomimicry of tumor cells and modulate expression of androgen-responsive genes in LNCaP cells	[50]

A number of the limitations of static cultivation schemes can be circumvented by using dynamic cultivation systems: i.e. BRs where the culture medium moves controllably.

## STIRRING BIOREACTORS

Stirring BRs (spinner-flask bioreactor, spinner vessel, stirred tank) represented a leap in improving the mass transfer between cells and the culture medium. These BRs are usually constructed as a tank vessel with a built-in rotating element, a spinner (long spatula), that forms vortex fluid flows, providing dynamic mixing of the medium and mass-transfer between the medium and tissue or scaffold. Stirring BRs also include systems where the movement of the medium around the scaffolds, tissues, or TECs is realized by the movement of the culture containers themselves. Examples include roller bottles and classical culture vessels placed on shaking, vortexing, or spinning automatic platforms (shakers). In stirring BRs, cell layers, tissue fragments, scaffolds or TECs are placed either on special needles or directly on the inner surfaces of culture vessels. In this case, the tissues/scaffolds can be completely immersed in the fluid occurring at the liquid-gas phase interface or alternately immersed into the culture medium and the gas phase.

Now, stirring BRs are mainly used to expand the cell mass (much more effectively, in comparison with a monolayer [51]), in particular in the form of cultures on microcarriers and as multicellular spheroids. The better nutrition of cells enables the production of larger spheroids [52]. Studies of the production of heterospheroids, which are co-cultures of tumor and normal cells, are of particular interest. For example, stirring BRs have been used to prepare hetero spheroids consisting of head and neck squamous carcinoma cells and peripheral blood mononuclear cells [53]. Study of the pharmacological effects of catumaxomab in spheroid models adequately reflects the properties of the micrometastases of these tumors. Spheroids derived from human brain tumor cells (glioma and astrocytoma) were seeded on porous scaffolds made from polylactic acid and cultured in multiwell plates on an orbital shaker under hypoxic conditions [54]. The cells in a 3Dmedium were found to acquire increased resistance to pro-apoptotic factors. Also, the hypoxia enhanced the resistance to cytotoxic drugs in monolayer cultures, although the molecular anti-apoptotic mechanisms in 2D- and 3D-cultures were different. A hybrid system “plate-on-a-shaker” was used to detect activation of angiogenic signaling pathways regulation and a decrease in the sensitivity of cells to chemotherapeutic anticancer drugs in 3D cultures placed in a complex scaffold made from poly(lactic-co-glycolic acid) and Matrigel [55]. The use of a stirring BR in an experiment with osteosarcoma cells provided compelling evidence of the advantages of complex TETMs on solid scaffolds (fibrous-bed) over tumor cell cultures on micro-carriers, apparently due to the reduced shear stress effects [56]. TECs composed of osteosarcoma cells and a porous fibrous scaffold proved stable in a culture for more than 1 month. On day 4, the cells stopped dividing but the fraction of apoptotic cells did not exceed 15%.

Stirring BRs are also used for DCL and RCL. DCL of small tissue fragments is usually processed in these BRs. In our recent paper [39], we demonstrated that systems like “flask-on-a-shaker” were feasible for the DCL of whole organs of laboratory animals. The efficiency of stirring BRs for the colonization and feeding of the cells was higher than that of static BRs, but it was still insufficient for TECs due to their relatively large sizes. In addition, the culture medium insufficiently penetrated into the construct; therefore, the cells distributed mainly over the scaffold periphery because of the diffusion limitations. The possibility of increasing the convection component with an increase of the rotation speed of the spinner or the culture vessel itself was limited due to shear-stress-induced tissue damage ( > 15 dyne/cm2) [51, 57].

The potential future applications of stirring BRs in TTE were not fully implemented, although these systems had a number of important advantages. These include adjustability of the culture volume, support of various TE models, availability of hydrodynamic computational models [34], accessibility for a sampling culture medium, and TEC state monitoring.

## ROTARY BIOREACTORS

Rotary BRs (rotating-wall bioreactors, NASA bioreactors; RWV; RCCSTM; HARV; STLV; RWPV), originally developed by NASA for experiments in space, are normally cylindrical containers with rotating walls completely filled with a culture medium. Horizontal (RWV) [58] and vertical (HARV) [59] rotary BRs revolve around a horizontal or vertical central axis, respectively, while oxygen is delivered through a stationary axial membrane or a similar membrane at the cylinder base. In these reactors, the culture medium is replaced manually via service openings. In a rotary BR with perfusion (RWPV or STLV), the culture medium circulates in a closed loop and is continuously replaced, which enables automatic maintenance of an optimal level of oxygen, pH, and temperature for many months. A RWPV consists of two cylinders, with the inner cylinder (which also serves as a gas exchange membrane) also able to rotate. The culture medium and the TECs are located in an annular space between the cylinders [60].

In rotary BRs, the scaffolds or TECs move freely in a culture chamber completely filled with the culture medium. The rotation speed of the cylinders (about 15–40 rpm) is adjusted to ensure balance between the gravity and the hydrodynamic resistance acting on the scaffolds/TECs, whereby the scaffolds/TECs are in a permanent state of free fall. The dynamic laminar (instead of turbulent, as in stirring BRs) flow of the culture medium can effectively bypass the diffusion limitations for the delivery of nutrients and removal of waste. Rotary systems provide a more uniform distribution of the cells compared to that in a static culture and better metabolism compared to that in stirring BRs. To compensate for the mass of growing tissue, the rotation speed is gradually increased in order to balance the gravity force and to ensure a suspended state of the TECs.

By using a rotary BR, differences in the effect of the 2D and 3D microenvironments on the expression of hepatocellular carcinoma genes were detected. HepG2 cells in multicellular spheroids, which reached a diameter of 100 μm in 72 h and 1 mm during a prolonged cultivation, exhibited increased expression of metabolic and synthetic genes, whereas activation of the genes encoding proteins of the extracellular matrix and cytoskeleton, as well as cell adhesion molecules, was observed in 2D. In addition, the liver cancer cells in spheroids retained a high activity of cytochrome P450 and produced albumin for a long time, whereas these features quickly degraded in a monolayer culture [61].

Interesting results were obtained in co-cultures of tumor and normal cells using a rotary BR. For example, colon adenocarcinoma cells (HT29 and HT29KM lines) formed spheroids in a monoculture, while in the presence of normal fibroblasts these cells competed with them for an attachment substrate and their growth was initially restricted. Then, the tumor cells began to divide actively and form bulky tissue masses of up to 1.5 cm in size that structurally resembled healthy intestinal crypts. A cell layer directly contacting the microcarrier’s surface was formed by young mesenchymal cells. Necrotic changes in these 3D cultures were almost absent [62]. Co-culture of breast cancer cells (UACC- 893, BT-20, and MDA-MB-453 lines) with fibroblasts in a rotary BR led to the formation of histoids, which are multicellular spheroids composed of fibroblasts with invading cancer cells [63]. Especially large heterospheroids (up to 1 cm in diameter) were produced in a HARV rotary BR from immortalized normal human skin keratinocytes HaCaT and cells of different melanoma lines (murine B16-F10 and human SKMEL-5) [64]. This biomimetic 3D model of melanoma was exploited to demonstrate a technique of cell transfection with plasmids encoding GFP and IL-15 which provided high reproducibility of the results of gene delivery. A rotary BR was also used to study the interaction among prostate cancer cells, osteocytes, and bone tissue cells in a 3D model [65].

An experiment on the production of spheroids from prostate cancer cells of different maturity using a rotary BR revealed significant differences in the spheroid spatial organization and proliferative activity, depending on the proportion of cell types [66]. According to the authors, this indicates the influence of cell differentiation on the spheroid packing density and, consequently, the efficiency of mass transfer between a cell aggregate and a culture medium.

Potential limitations in the use of rotary BRs are associated with the generation of laminar fluid flow shear stress (in the range of 0.5–2 dynes/cm2) [67]. The efficiency and safety of rotary BRs can be improved by combining them with reactors operating on other principles [68].

## PERFUSION (FLOW) BIOREACTORS

Perfusion bioreactors allow one to perform the most accurate reproduction of the mass transfer processes in a living organism. A typical perfusion BR consists of a pump and an incubation chamber connected by flexible tubes to form a system with an open or closed loop. The pump creates a slight overpressure, providing a permanent liquid medium flow through tissues and scaffolds. Perfusion BRs can be used both for DCL and for RCL. In perfusion DCL, solutions of detergents or other substances promoting deattachment, destruction, and removal of the cells are delivered through natural blood vessels connected to a perfusion contour.

During RCL, a cell suspension is transported through the decellularized vascular conduits of the treated tissue/ TEC or through other voids in the scaffold. This provides a more homogeneous distribution of cells in the matrix and better transport of liquids compared to those in stirring and rotary devices [69-72]. As a result, long-term growth and maintenance of larger TECs becomes possible [72, 73]. The survival rate of the cells seeded on the scaffolds perfused by means of these BRs is substantially higher compared to that in a static culture or stirring BR [74]. Regulation of the medium flow rate in BRs enables to control both the shear stress associated with the fluid flow and the local distribution of oxygen across a TEC [75]. At the same time, although perfusion BRs definitely can improve the control of mass transfer, in comparison with the other systems, the problem of non-uniform delivery of the necessary substances has still not been completely resolved. This is especially notable for scaffolds with pores of widely varying sizes and also for tissues with a non-uniform growth rate, which results in insufficient nutrition of some areas and excessive delivery to others [70].

Perfusion BRs are of critical importance to wholeorgan tissue engineering – a formation of whole-organ TECs [30, 76] using sequential DCL and RCL processes. These reactors are subjected to particularly strict requirements to ensure control over the parameters of the culture medium/cell suspension flow, sterility, temperature, and the possibility to monitor organ treatment or organ TEC formation [76-80].

Perfusion BRs are actively used for the reconstruction of normal tissues and organs, but their application in the development of TETMs has just started. For example, a colorectal cancer model was demostrated using a commercially available perfusion BR [81]. HT- 29 line cells were conventionally cultured in a monolayer or seeded on collagen sponges and maintained in a static 3D culture (as the control samples) or in a perfusion BR. Additional control was provided with tumor xenografts implanted in athymic mice using the same cell line. The cells in the perfusion culture were characterized by a much higher proliferation rate and a much more uniform distribution compared to those in the static bulky culture. The produced TECs were morphologically and phenotypically similar to the tumors developed from the implanted cells. A strong correlation between perfusion 3D cultures and tumor xenografts was also observed in the expression profiles of the genes that regulate apoptosis and the response to hypoxia. Comparison of the effects of 5-fluorouracil and ABT-199, an inhibitor of the anti-apoptotic gene* BCL-2*, showed a fundamental difference in the cellular responses both in 2D and in 3D TECs. The same paper described a preparation of TECs on collagen scaffolds using cells of colorectal (SW480 and DLD-1), prostate (PC-3), non-small cell lung (A549), and breast (BT-474) cancers.

A sophisticated TE model of schwannoma (neurofibrosarcoma) was developed by German researchers using a specially designed BR [82]. An isolated porcine intestinal fragment was subjected to alternating perfusion DCL through the mesenteric artery and lumen of the intestine and to the immersion DCL on a shaking platform. The resulting matrix was sterilized by gamma radiation. Then, the DCL matrix of the intestine fragment was cut along the long axis and the resulting membrane was stretched between two metal rings and placed in a perfusion BR chamber. The scaffold was seeded with the primary skin fibroblasts and linear tumor cells of schwannoma S462 (on the apical surface) and microvascular endothelial cells (on the basolateral surface of the intestinal segment). The TEC was incubated in a perfusion culture at a permanent or pulsed flow of the culture medium for about 2 weeks.

Recently, the technologies of perfusion DCL and RCL of organs were used to generate a TE lung cancer model [83]. Different types of linear lung cancer cells (A549, H460, H1299) were seeded by perfusion of the cell suspension on a decellularized whole-organ scaffold prepared from mouse lungs. Then, the whole-organ TECs were perfused with an oxygenated culture medium and maintained *ex vivo *for up to 2 weeks. The authors demonstrated the formation of macroscopic tumor nodes with their own vasculature, the development of typical cell-cell and cell-matrix interactions, and the formation of a typical structure and dynamics of tumor growth similar to those of real fragments of human lung cancer tissue.

## HOLLOW-FIBER BIOREACTORS

A hollow-fiber BR is a closed vessel filled with a cell suspension in a culture medium or a scaffold or (potentially) a complex TEC permeable to the medium. This scaffold/TEC contains a bundle of mutually parallel semi-permeable hollow fibers mimicking blood vessels and providing delivery of the nutrients to the cells and the removal of waste. The main advantage of these BRs is their ability to deliver nutrients in the depth of the growing engineered tissues. Hollow-fiber BRs are successfully used in experiments on the culture of very sensitive cell types with a high metabolic demand, such as hepatocytes [84], the attempts to use a similar system to create 3D constructs have not been successful. It turned out that the high densities of the cellular suspensions or solid matrices significantly limit mass transfer and oxygen diffusion in this system. This leads to the death of cells at longer distances from the hollow fibers and to the loss of the structural homogeneity of tissue [85]. To solve this problem, a coaxial BR design based on hollow fibers inserted into each other and forming independent compartments for growing cells was proposed [84, 86]. The coaxial design significantly improved mass transfer. However, another serious drawback of these systems is the inability to avoid damage to the formed tissue during extraction of a TEC from the BR for further use.

Hollow-fiber BRs were used in experimental oncology to expand cell mass, obtain the specific cell products, and monitor the tumor tissue metabolism. For example, T-cells isolated from an inflammatory infiltrate of ovarian cancer biopsies were cultured in a hollow-fiber BR [87]. A commercially available BR was used to produce spheroids from breast cancer cells (MCF-7) and to study the effects of δ-tocopherol concentrations [88]. As a result, a technique based on contrast-enhanced MRI was proposed for monitoring the cell density and oxygen concentration in spheroids [88]. Also, MRI and a hollow-fiber BR were used to determine the mechanism of changes in the apparent diffusion coefficient of water (an important diagnostic sign) in a ischemic brain tissue that was simulated using a 3D culture of rat glioma cells [89, 90].

## MICROFLUIDIC BIOREACTORS

Microfluidic platforms (microfluidic chips, microfluidic bioreactors) can be considered as a special kind of perfusion BR scheme for the development and study of biological objects consisting of about 102–103 cells. By using a multistep technology, a glass substrate is covered with a layer of biocompatible silicone material (polydimethylsiloxane) arranged as microchannels and microcontainers. The advantage of this polymer over polystyrene (conventionally used in cell culture) is a combination of high permeability to oxygen and to other gases with almost complete impermeability to water [91]. In microfluidic BRs, mass transfer to cells that grow in hydrogel-filled microwells or directly on the chip elements occurs by perfusion of a culture medium through the microchannels.

The variability and adaptability of microfluidic systems can help solve very different problems and contribute to the active development of organ(s)-on-a-chip and lab-on-a-chip technologies. An important advantage of these BRs is precise control over the parameters of culture medium flows and optical imaging *in situ *in real time [92]. Microfluidic systems are used to study the cell responses to the action of signaling molecules, as well as the effects of metabolic and physical gradients and the role of interstitial fluid flows in the metabolism of tissues, including tumors. They also are very useful for precise quantification of the permeability of TECs to drugs and nanoparticles [28, 93-96]. Furthermore, these systems can be used to simulate the kinetics of cell populations, progression of tumors, angiogenesis, invasion and other stages of metastasis [97-103].

## BIOREACTORS WITH A DIRECT IMPACT ON A SCAFFOLD/TEC

BRs also can provide a direct controlled action of various physical factors on a scaffold or TEC. For example, a TE object can be exposed to mechanical forces, electrical impulses, or different types of radiation. The most progress has been achieved in the bioreactor technologies associated with biomechanical research.

## COMPRESSION BIOREACTORS

Compression BRs are widely used in tissue engineering, especially in the preparation of cartilage structures. These BRs consist of an engine, a system providing linear displacement, and a control mechanism. The stress is usually transmitted to a cell-seeded scaffold through flat rollers [104] and exerts a specific mechanical effect on cells and an increased fluid flow through a TEC. In TTE, compression BRs can be used, in particular, to simulate the mechanisms of bone metastatic niche formation. Currently, almost nothing is known about the response of metastatic cancer tissue to mechanical stress [105]. A study on 3D cultures of breast cancer (MDA-MB-231) and glioblastoma (U87, HGL21) cells in a compression BR demonstrated increased expression of the genes responsible for enzymatic lysis of extracellular matrix proteins, as well as adhesion and migration in response to increased static compression. This corresponded to an increase in the metastatic potential [106].

## STRAIN BIOREACTORS

BRs with controlled mechanical strain (strain bioreactors) are structurally similar to compression BRs and differ only in the way the stress to a sample is transmitted. Scaffolds/TECs are secured in such a way that the straining force can be applied to them. For example, they are placed on a rubber membrane that is then deformed [107]. In TTE, a model was recently proposed to study the role of mechanical tension of the extracellular matrix in the induction of an invasion of 3D organoids produced by culturing transformed epithelial cells in collagen gels of different concentrations. A gel incorporating cell aggregates and covalently bound to a polydimethylsiloxane base was placed in a microfluidic chip with a device for straining that part of the culture chamber. A positive correlation between the invasiveness of the cells and the gel stiffness was found; a concentration effect associated with changes in the mean pore size was revealed [108].

## HYDROSTATIC PRESSURE BIOREACTORS

In hydrostatic pressure BRs, mechanical compression on the scaffolds or TECs is implemented through a periodic reduction in the culture chamber volume, while the culture medium volume remains constant [109]. These BRs are not extensively used in TTE; however, this direction seems promising only for the modeling of one of the most important physiological features of solid tumors such as the increased interstitial pressure [36].

## BIOREACTORS FOR ELECTRICAL STIMULATION OF CELLS AND TISSUES

BRs with electrical stimulation are usually used for the modeling of excitable tissues. According to our data, there has been no mass use of such BRs for the development of cancer 3D models, but there are a few reports on the culturing of tumor cells in hydrogel under weak electric field conditions (at an electric field intensity of 1.1 V/cm and a variable frequency of 150 kHz and 200 kHz) in a hybrid microfluidic chip-based device [110]. The authors observed changes in the morphological characteristics of lung (A549) and breast (MDA-MB-231) linear cancer cells, a decreased proliferation rate of both tumor cell lines, and signs of a reduced metastatic potential of A549 cells. At the same time, the electrical stimuli did not alter the activity of normal human endothelial cells (HUVEC).

## COMBINED BIOREACTORS

Numerous BR combinations have been developed that allow one to grow tissues under laboratory conditions that are set maximally close to natural ones. Usually, these combinations include adding various methods of mechanical impact on a tissue to a standard perfusion or rotary BR. For example, combining a strain BR, hydrostatic pressure BR, or compression BR with a perfusion or rotary BR combines the advantages of improved mass transfer by perfusion or rotation and mechanical stimulation of TECs.

## CONCLUSION

Bioreactor technologies adapted for biomimetic models of malignant tumors were discussed and critically analyzed in this paper.

Static systems and stirring BRs designed on the basis of conventional culture vessels placed on shakers remain the most commonly used in tumor tissue engineering (TTE). At the same time, a number of important developments are emerging. In particular, microfluidic systems representing a promising TTE platform. It becomes clear now that the best mimicking of the key properties of cancers calls for a multimodal biological reactor (BR), which represents a hybrid of the existing modalities addressed in this review. A new-generation BR is envisaged as a multipurpose device with automated control over tissue engineering processes and augmented standardization of cultivation conditions. This BR could provide one of the gateways to the elucidation of cancer biology. At the same time, BR-based experiments open conceptual possibilities for testing prospective generations of anticancer agents based on recombinant molecules [111-118], multifunctional nanostructures [119-126], as well as the evaluation of new cell and tissue engineering technologies [25, 39-41, 127, 128] for the reprogramming of cancer cells [129, 130].

## Abbreviations

2D: two dimensional/monolayer cell or tissue culture in vitro
3D: three-dimensional
BR: bioreactor
DCL: decellularization
DCL matrix: decellularized matrix
DCL tissue: decellularized tissue
DCL organ: decellularized organ, respectively
RCL: recellularization
TE: tissue engineering
TEC: tissue engineering construct
TTE: tumor tissue engineering
TETM: tissue-engineered tumor model
SCID: severe combined immunodeficiency mice
RWV: rotating-wall vessel (rotating-wall bioreactor)
RCCS: rotary cell culture system
HARV: high aspect reactor vessel
STLV: slow turning lateral vessel
RWPV: rotating-wall perfusion vessel
NASA bioreactor: common name of rotary bioreactors developed by NASA, usually RWW, HARV, or STLW
